# Dietary Inflammatory Score (DIS)’s and Lifestyle Inflammatory Score (LIS)’s Impact on Multiple Sclerosis Severity

**DOI:** 10.3390/nu17030526

**Published:** 2025-01-31

**Authors:** Monica Guglielmetti, Cinzia Ferraris, Lenycia de Cassya Lopes Neri, Evelyn Frias-Toral, Anna Tagliabue, Eleonora Tavazzi, Alessandro La Malfa, Giacomo Greco, Roberto Bergamaschi, Raynier Zambrano-Villacres, Giuseppe Grosso

**Affiliations:** 1 Human Nutrition Center, Department of Public Health, Experimental and Forensics Medicine, University of Pavia, 27100 Pavia, Italy; monica.guglielmetti@unipv.it (M.G.); lenyciadecassya.lopesneri@unipv.it (L.d.C.L.N.); 2 Food Education and Sport Nutrition Laboratory, Department of Public Health, Experimental and Forensics Medicine, University of Pavia, 27100 Pavia, Italy; 3 School of Medicine, Universidad Católica de Santiago de Guayaquil, Av. Pdte. Carlos Julio Arosemena Tola, Guayaquil 090615, Ecuador; evelyn.frias@cu.ucsg.edu.ec; 4 Multiple Sclerosis Center, IRCCS Mondino Foundation, 27100 Pavia, Italy; eleonora.tavazzi@mondino.it (E.T.); alessandro.lamalfa02@universitadipavia.it (A.L.M.); giacomo.greco@mondino.it (G.G.); roberto.bergamaschi@mondino.it (R.B.); 5 Escuela de Nutrición y Dietética, Universidad Espíritu Santo, Samborondón 0901952, Ecuador; rzambranovillacres@uees.edu.ec; 6 Department of Biomedical and Biotechnological Sciences, University of Catania, 95123 Catania, Italy; 7 Center for Human Nutrition and Mediterranean Foods (NUTREA), University of Catania, 95123 Catania, Italy

**Keywords:** dietary habits, lifestyle, multiple sclerosis, inflammation

## Abstract

**Background/Objectives**: Multiple sclerosis (MS) is an immune-mediated demyelinating disease of the central nervous system with variable degrees of inflammation and gliosis. Diet and lifestyle factors could influence MS development and also contribute to inflammation. The current study aims to evaluate the relationship between dietary and lifestyle inflammatory potential and multiple sclerosis severity. **Methods:** A cross-sectional study design was employed. Data collection included demographic, neurological, and nutritional information. The Dietary Inflammatory Score (DIS) and Lifestyle Inflammatory Score (LIS) were calculated based on the reference protocol. **Results:** One hundred and seven participants (69.2% female; mean age, 50.6 ± 11.6 years) completed the study. The anti-inflammatory LIS group had significantly higher proportions of normal-weight (*p* = 0.000) and physically active (*p* = 0.022) participants. A greater proportion of women exhibited an anti-inflammatory lifestyle compared to men (80% vs. 20%; *p* = 0.023). No relation was retrieved between the DIS, LIS, and MS Severity Score (MSSS). When analyzing the single DIS components, leafy greens were associated with MS severity (OR 1.67; 95% CI, 1.50–18.74; *p* = 0.009). Among the LIS components, “high physical activity” (OR 5.51; 95% CI, 1.66–18.30; *p* = 0.005) and “heavy drinking” (OR 5.61; 95% CI, 1.19–26.47; *p* = 0.029) were related to lower MS severity. **Conclusions**: Although no differences were found in the total Dietary and Lifestyle Inflammatory Scores, some of their components might be connected with MS severity. Further intervention studies are needed to validate these findings.

## 1. Introduction

Inflammation is characterized by an increase in pro-inflammatory markers (e.g., cytokines or C-reactive protein) in tissues and/or in the blood as part of the immune system’s response to external stimuli considered potentially harmful to the organism. It becomes a chronic state when prolonged due to autoimmune disorders, frequent episodes of acute inflammation, failure to remove the external agent, or unresolved injuries [1,2]. Low-grade inflammation has been linked to the development of various diseases, such as obesity, metabolic syndrome [3], cancer [4], cardiovascular diseases [5], and neurodegenerative conditions [6]. Among the latter, multiple sclerosis (MS) is an immune-mediated demyelinating disease of the central nervous system (CNS) that involves varying degrees of inflammation and gliosis [7]. It contributes to neurodegeneration, emerging as a leading cause of progressive disability and premature retirement among young adults [8]. The causes of the inflammatory state typical of MS are uncertain but probably derive from a combination of several determinants of genetic, autoimmune, and environmental origin [9,10,11].

Diet has been reported as an important environmental factor that could influence MS development and also contribute to inflammation [12,13]. At the molecular level, some nutrients, upon binding to their target, can activate signal transduction mechanisms that are involved in the immune system and inflammation [14]. Nutrients also activate specific transcription factors that regulate the expression of genes related to metabolism, including nutrient metabolism and energy homeostasis, resulting in the activation of anabolic or catabolic pathways [15]. In fact, foods typical of Western regimens (e.g., processed meats, fried foods, sugary drinks, and sweets) have been associated with an increase in C-reactive protein levels and pro-inflammatory cytokines [16,17,18] and thus defined as pro-inflammatory themselves. Conversely, a diet rich in vegetables, fibers, polyphenols, alfa-lipoic acid, and essential fatty acids (omega-3), alongside other interventions such as caloric restriction and physical exercise, displace the metabolic balance towards catabolic pathways, leading to anti-inflammatory effects [19,20,21,22].

Although the impact of individual dietary components on inflammation is probably limited and unlikely to provide protection per se, whole dietary patterns have been associated with a lower burden on the immune system and inflammatory biomarkers. In this context, individuals adopting plant-based dietary patterns, such as the Mediterranean diet, have been reported to reduce the risk of MS or to be associated with less severe symptoms [23]. As far as Dietary Inflammatory Scores are concerned, previous studies have shown a positive association between the pro-inflammatory potential of a diet and the likelihood of developing MS [24]. However, no previous instrument was able to assess the impact of lifestyle factors on inflammation. A double score, the Dietary Inflammatory Score (DIS) and the Lifestyle Inflammatory Score (LIS) developed by Byrd et al. [25], aims to identify both dietary and lifestyle characteristics based on biological plausibility from the prior scientific literature on the anti- and pro-inflammatory potential of various constituents. These scores, verified through comparison with inflammatory markers, have been associated with numerous conditions, such as anxiety and depression [26], diabetes [27], metabolic syndrome [3], or chronic kidney disease [28].

To the best of our knowledge, no previous studies have examined the impact of lifestyle factors on MS severity, despite evidence suggesting that smoking, the body mass index (BMI), alcohol consumption, and physical activity may play significant roles [29]. Therefore, the current study aims to evaluate the relationship between dietary and lifestyle inflammatory potential and MS severity, expressed by the Multiple Sclerosis Severity Score (MSSS).

## 2. Materials and Methods

A cross-sectional study was designed and performed by the Human Nutrition and Eating Disorders Research Center—University of Pavia in collaboration with the C. Mondino National Neurological Institute, Pavia, Italy. Ethical approval was obtained by the San Matteo Ethical Committee (P-20200064205, date: 5 August 2020). Due to restrictions during the COVID-19 pandemic, in-person visits were not feasible, and data were collected through a telephonic interview (mean duration: 30–40 min). This protocol adhered to the Declaration of Helsinki.

Recruitment was conducted by a neurologist during routine control visits. Individuals who agreed to participate were telephonically contacted by the dietitian to schedule the interview appointment, which was performed in the days immediately after the neurological visit (range 3–10 days). Unfortunately, it was not possible to carry out the interview in person, as explained before. Participants were asked to review and sign the privacy policy prior to study initiation. The protocol and procedures have been presented in previous studies [30,31]. MS diagnosis was performed according to the McDonald criteria [32]. No restriction was made in the inclusion criteria based on the MS phenotype or level of disability.

### 2.1. Data Collection

Demographic information included age, gender, educational level, and marital status. Lifestyle information focused on smoking and physical activity habits, while alcohol consumption was investigated with the administered food frequency questionnaire (FFQ).

Basic anthropometric information, including body mass (BM, in kg) and height (in meters), was self-reported by the participants in the telephonic interview. The BMI was subsequently calculated and classified into underweight (BMI < 18.5 kg/m^2^), normal weight (18.5 < BMI ≤ 24.9 kg/m^2^), overweight (25 ≤ BMI ≤ 29.9 kg/m^2^), and obesity (BMI ≥ 30 kg/m^2^) [33]. An expert dietitian collected the participants’ dietary habits from the previous six months, using a validated FFQ [34]. Neurological data collection included the level of disability according to the Expanded Disability Status Scale (EDSS) [35], the disease duration and severity (MSSS) [36], current therapies, and treatment types.

### 2.2. Food Frequency Questionnaire

A validated food frequency questionnaire (FFQ) [34] was applied to assess the patients’ dietary habits. The questionnaire was made up of 110 elements, investigating nine different food groups (meat and fish, sweets and snacks, dressings, vegetables, fruit, drinks, cereals and bread, dairy, dietary supplements, and herbs). For each item, the individual was asked to declare the mean frequency of consumption during the previous six months. The patients were individually contacted and telephonically interviewed by a skilled dietician (about 30–40 min). The interviewee asked how frequently on a monthly (“never”, “once”, or “twice”), weekly (“once”, “two or three times”, or “four or five times”), or daily (“once”, “two or three times”, or “four or five times”) basis the participant habitually consumed each specific food. For most of the food included in the FFQ, a specific portion size was indicated (e.g., 100 g of fish). If the amount of food consumed was different from the reported portion, the size was adjusted. Thus, the food frequencies reported by each individual were transformed into daily intakes for each item and their mean values were calculated (in g or mL). Energy and macro- and micronutrient intakes were computed by referring to the standard food composition tables of the Italian Research Center for Foods and Nutrition. Food seasonality was taken into account by asking the patient to report the food’s frequency of consumption during the span in which it was accessible; then, it was harmonized with the whole year.

### 2.3. Dietary and Lifestyle Inflammatory Scores

The DIS and LIS were calculated according to Byrd et al. [25]. The DIS was made up of 19 elements, comprising 18 food items or groups (e.g., coffee and tea, leafy green vegetables, high- and low-fat dairy products) and sixteen single micronutrient supplements used (e.g., vitamins A, B-12, β-carotene, folate, calcium, magnesium, zinc, iron). According to Byrd et al. [25], the DIS did not incorporate soy and whole grains due to insufficient intake in the reference cohort and inadequate measurement, respectively. Food recipes (e.g., those for pizza) were disassembled into individual components and normalized to 100 g portions before classification into relevant food groups. A weighted positive or negative score was given to each component according to its pro-inflammatory or anti-inflammatory properties, respectively. The LIS was composed of four elements: the BMI, smoking habits, physical activity, and alcohol consumption. Each constituent was further divided into subclasses as follows: (a) alcohol consumption: moderate drinkers (less than 1 drink/day for women and less than 2 drinks/day for men) and heavy drinkers (more than 1 drink/day and 2 drinks/day for women and men, respectively); (b) smoking habits: never smoked, current smoker, and former smoker; (c) BMI: normal weight/underweight, overweight, and obesity (only overweight and obesity were considered in the final LIS, as pro-inflammatory factors); physical activity: low activity (0 times per week), moderate activity (1–3 times per week), and high activity (≥4 times per week). Higher DISs and LISs indicated higher pro-inflammatory potential. The participants were divided into “anti-inflammatory” (DIS < 0) and “pro-inflammatory” (DIS > 0) groups. The same criterion was applied to the LISs.

### 2.4. Neurological Information

MS disability was estimated using the Expanded Disability Status Score (EDSS) [35], which represents the most commonly used and internationally validated tool in clinical practice to evaluate the level of impairment determined by the disease. It includes a multifunctional clinical assessment of all the systems usually evaluated in clinical practice (e.g., the visual, brainstem, pyramidal, cerebellar, sensory, cerebral, and sphincter systems). Disease duration, expressed in years, was calculated and reported as means and standard deviations. Based on the disease duration and EDSS, the Multiple Sclerosis Severity Score (MSSS) [37] was computed for each individual. Disease-modifying therapy (DMT) use was assessed (yes/no) and, if present, was grouped into “moderate efficacy” or “high-efficacy”.

### 2.5. Statistical Analysis

The frequencies of occurrence and percentages were used to express categorical variables, while the Chi-squared test was used to determine the differences between the DIS and LIS groups. The DIS and LIS were considered categorical variables (pro-inflammatory and anti-inflammatory). The mean and standard deviations (SDs) expressed quantitative variables, with Student’s t-test used to explore differences between the MS severity and DIS and LIS groups. Associations between the level of adherence and MS severity were assessed through logistic regression analyses and odds ratio (OR) calculations with respective 95% confidence intervals (CIs). Unadjusted and multivariate models adjusted for the confounders (energy intake, age, sex, BMI, educational status, smoking status, and physical activity level) were calculated. *p*-values were based on two-sided tests and aligned with a significance level of 5%. SPSS 21 (SPSS Inc., Chicago, IL, USA) software was used for the statistical analysis.

## 3. Results

One hundred and thirty participants were recruited. Of these, twenty-four were excluded due to missing data (n = 19) and objection to completing the telephonic interview after enrollment (n = 5). One hundred and seven patients with multiple sclerosis (pwMS) composed the final sample (mean age, 50.4 ± 11.6 years). Most participants were female (69.2%), of a normal weight (66.4%), and non-smokers (61.7%). Of the sample, 40.2% had a low physical activity level, and 20.6% had a low educational level. Regarding neurological information, the mean MSSS was 3.2 ± 2.4. The relapsing–remitting phenotype was the most frequent (72.0%), and 66.4% of patients were undergoing therapy, with first-line therapy being most commonly used (74.3%).

Table 1 further describes the sample’s baseline characteristics according to the DISs and LISs. No baseline differences were recorded between the anti-inflammatory and pro-inflammatory DIS groups. However, the LIS groups expressed significant differences in the sex distribution (*p* = 0.023), BMI (*p* < 0.001), and physical activity levels (*p* = 0.022). A trend toward significance was also retrieved in smoking habits (*p* = 0.063). No significant differences concerning disease-related factors (medication use, MS type, and disease duration) were detected (Table 1).

The nutritional composition of the participants’ dietary habits is summarized in Table 2. No significant differences in energy or macronutrients were found between DIS groups, except for fiber intake (*p* < 0.001). Moreover, the saturated fatty acid intake was about 2.5 times above the recommended reference intake [38] in all the analyzed groups, but there were no significant differences. The overall total fat, MUFA, and PUFA intakes were within the reference range. As for micronutrients, a statistically significant difference was found in vitamins A, C, E, and D, riboflavin, niacin, potassium, iron, calcium, copper, zinc, phosphorus, and magnesium. No differences in micronutrient intake were reported between LIS groups.

A total of 16 (35.6%) patients with an anti-inflammatory DIS profile had mild MS, while 29 (64.4%) had moderate to high MS (*p* = 0.372). There were also no statistically significant differences concerning patients with an anti-inflammatory DIS profile [20 (40%) mild vs. 30 (60%) moderate to high; *p* = 0.963]. Linear regression analysis failed to demonstrate a relation between the DIS and LIS in the MSSS (for DIS: beta = −0.181; SE = 0.108; and *p* = 0.098; for LIS: beta = −0.315; SE = 0.368; and *p* = 0.934). Nonetheless, when analyzing the single DIS components, leafy greens were associated with MS severity (OR 1.67; 95% CI: 1.50–18.74) (Table 3).

Among the LIS constituents, significant associations were found between heavy drinkers and mild MS (OR 5.61; 95% CI: 1.19–26.47) and high physical activity (OR 5.51; 95% CI: 1.66–18.30) (Table 4). A trend toward significance was also retrieved for obesity (OR 6.38; 95% CI: 0.94, 43.48)

## 4. Discussion

Low-grade chronic inflammation plays a central role in the pathophysiology of MS [6], with diet and lifestyle potentially contributing not only to the progression or improvement of the inflammatory state but also to the onset and course of the disease. Our study aimed to assess whether the DIS or LIS were related to MS severity, expressed by the MSSS. Based on our results, the DIS and LIS were not predictors of MS severity, but, when analyzing the sample’s characteristics, their dietary habits, and the single constituents of the DIS and LIS, interesting significant associations were retrieved.

Regarding baseline features, a significant association was found between the LIS and sex, the BMI, and physical activity. The distribution of BMI categories was significantly different within the two LIS groups: the anti-inflammatory one had a higher percentage of normal weight compared to the pro-inflammatory one (88% vs. 47.4%), with the remaining participants classified as overweight (12.0% vs. 40.4%). No cases of obesity were reported in the anti-inflammatory group, whereas 12.3% of participants in the pro-inflammatory group were classified as individuals with obesity. The BMI is one of the four major components of the LIS, and the link between obesity and low-grade inflammation is well documented [39,40]. Individuals with obesity, in fact, often present significantly higher concentrations of pro-inflammatory markers, with up to a 10-fold increase compared to normal-weight subjects [39,41]. Among the various contributing factors, adipose tissue might be a relevant determinant of inflammation [42], particularly through the pro-inflammatory and anti-inflammatory capacities of adipokines (e.g., adiponectin and leptin) [43,44]. Notably, this low-grade inflammation can be reversible with weight loss [32]. Moreover, obesity is often linked to a sedentary lifestyle. A recent review and meta-analysis involving 111,851 individuals with obesity [45] pointed out a significant increase in sedentary behaviors and physical inactivity among subjects with obesity (+31% and +43%, respectively). All these mechanisms can, at least partially, explain the BMI differences associated with the LIS.

A significant difference was also found in physical activity levels between the LIS groups. The anti-inflammatory group was characterized by a higher proportion of physically active individuals (38.0% vs. 17.5%) and a lower percentage of inactivity (28.0% vs. 50.9%), while the moderately physically active proportions were comparable. Adequate physical activity levels may exert anti-inflammatory effects by decreasing serum concentrations of interleukins, C-reactive protein, and other inflammatory markers [46], reducing fat mass (particularly the volume of adipose tissue cells) [47], improving cardiometabolic health and antioxidant capacity, and lessening vascular wall inflammation [48]. Additionally, higher physical activity is also linked to a decreased probability of obesity. For these reasons, moderate (one to three times/week) and high (at least four times/week) physical activity were included in the anti-inflammatory constituents of the Lifestyle Inflammatory Score. Therefore, it is not surprising that higher rates of physical activity were retrieved in the anti-inflammatory LIS group.

A greater proportion of women exhibited an anti-inflammatory lifestyle (80% vs. 20%), compared to a less marked gender distribution in the pro-inflammatory group (59.6% vs. 40.4%). To the best of our knowledge, no previous study has deepened the possible gender differences in the Lifestyle Inflammatory index; thus, a comparison of the results appears difficult. However, other studies have reported that women tend to have a healthier overall lifestyle in comparison to men. For example, Chang et al. (2019) [49], in a cross-sectional study examining the lifestyle habits of 1066 individuals in Taiwan with metabolic syndrome, highlighted greater levels of moderate-to-vigorous physical activity, lower smoking and obesity rates, and healthier dietary regimens in females. Similarly, a Brazilian cross-sectional study on a wide cohort of 15.000 employees described an overall healthier lifestyle in women, particularly after retirement. According to another recently published study [50], there are gender differences in lifestyle parameters, such as smoking habits, alcohol consumption, and physical activity, with a greater proportion of females falling in the “never smoking” and “no alcohol consumption” categories.

Overall, patients’ dietary intakes met the recommended nutritional needs for the Italian population, except for saturated fatty acid (SFA), sodium, and calcium intakes. SFA and sodium consumption went beyond the daily reference intakes, while calcium levels were slightly lower. Although not significant, the SFA intake deserves to be mentioned as particularly exceeding the Italian reference intake [38]. As already shown by Drehmer et al. (2020) [51], an imbalance was highlighted in the percentage distribution of MUFAs, PUFAs, and SFAs, with an excessive intake of saturated fatty acids. This is particularly important because SFAs can promote the maintenance of the chronic state of inflammation that characterizes the pathology. In fact, they represent one of the most studied pro-inflammatory factors, together with trans fatty acids, cholesterol, and cow’s milk proteins, which induce a shift in metabolic balance towards anabolism related to inflammation [15]. Saturated fats can help maintain a state of chronic systemic inflammation. Their consumption is also associated with increased LDL cholesterol, with an alteration in inflammatory responses [52,53]. Trans fatty acids, which often are associated with SFAs, are also positively associated with one intestinal inflammatory state and the hyperproduction of pro-inflammatory cytokines [54]. All these mechanisms can, at least partially, contribute to disease onset and progression. When considering the DIS and LIS classes, no significant differences in energy and macronutrient intake were found in the DIS groups, except for fiber, nor were any found in the LIS groups. As for micronutrients, a statistically significant difference was found in several vitamins and minerals according to the different DIS groups. Higher intakes of vitamins A, C, E, and D, riboflavin, niacin, potassium, iron, calcium, copper, zinc, phosphorus, and magnesium were found in the anti-inflammatory DIS group. No micronutrient intake differences were reported in the two LIS groups. In a cross-sectional study conducted with 265 healthy individuals aged 18–70 years, a greater DLIS (DIS and LIS combined score) was linked to higher intakes of fiber, energy, and macronutrients. This was attributed to the increased consumption of fruits, vegetables, meat, coffee, tea, high-fat dairy products, and refined cereals [55]. Most of these food groups are ascribed to anti-inflammatory foods [56], whereas processed meats, fried foods, soft drinks, sugar, and sweets can contribute to the inflammatory response [57]. Moreover, a cross-sectional study on pwMS revealed that a healthier dietary regimen, with high intakes of riboflavin, vitamin D, zinc, linolenic acid, vitamin C, β-carotene, and vitamin A, was associated with protective effects against MS [58]. Increased consumption of vegetable-origin foods, including fruits and vegetables, has been found to be positively related to lower odds of developing MS. In our study, leafy greens were associated with a 67% higher probability of mild multiple sclerosis. Similar results were found in a case–control study evaluating the effect of the Mediterranean-DASH Intervention for Neurodegenerative Delay (MIND) diet in 77 cases with relapsing–remitting MS and 148 healthy controls. Overall adherence to the MIND diet was related to a decreased risk of MS, with significantly lower odds of MS in the group with higher intakes of leafy green vegetables (OR = 0.02; 95% CI = 0.00–0.21; *p*-value < 0.001) and other vegetables [59]. One of the possible explanations is that leafy greens are rich in various antioxidants (e.g., β-carotene, glucosinolates, isothiocyanates, lutein) as well as flavonoids and polyphenols. As previously reported, polyphenols have, among others, important antioxidant and anti-inflammatory properties, which are essential for cellular protection against oxidative stress and inflammation [60]. According to a recent meta-analysis, leafy green vegetables have also been shown to have multiple health benefits, such as reductions in all-cause mortality, tumor incidence, and metabolic and cardiovascular risks [61].

As previously mentioned, the overall DISs and LISs were not associated with MS severity. The presence of pharmacological treatment in the majority of patients needs to be taken into account when interpreting the results, as the relevant anti-inflammatory effect of disease-modifying treatments might overcome and make null the effect of food on inflammation. Forty-four (66.4%) patients were not using treatment, but a statistical analysis of this subpopulation was not feasible for the heterogeneity of the sub-sample, represented by different MS subtypes, disease durations, and pharmacological histories, with 31.8% of the patients previously treated with different types of immunomodulating drugs.

However, a previous study investigating the inflammatory potential of diet showed that higher scores indicating a pro-inflammatory diet were associated with an increased risk of MS in 68 cases and 140 controls from Iran [62]. More recently, another study confirmed these findings in a wider population (547 new MS cases and 1057 controls), suggesting a greater risk of MS in those with increased scores [63]. The discordant results may depend on the different instruments used to estimate the inflammatory potential of the diet and the limited number of patients included in our study. Hence, further research is needed in this context with the enrollment of larger samples and the use of multiple tools to better assess inflammatory factors related to diet. Similarly, although no previous studies have used the LIS in the context of MS, other studies have highlighted the importance of a healthy lifestyle in MS [64,65]. For example, a combination of healthy lifestyle behaviors (i.e., no smoking, a healthy diet, regular physical activity, and maintaining a BMI under 30 kg/m^2^) was associated with a 71% reduced risk of MS [66]. In our study, only alcohol consumption and physical activity were significantly associated with a 5.5-fold higher likelihood of mild MS. Alcohol consumption has historically been investigated as one of the potential detrimental environmental factors related to MS, although moderate intake could present anti-inflammatory properties [67]. Some hypotheses about the potential mechanisms through which ethanol could negatively affect MS include its direct pharmacological effect on the CNS [68] and, in the long term period, the detrimental effects on executive function and loss of cerebral matter [69]. However, other studies have reported contrasting results when considering low-to-moderate alcohol consumption and MS disability. In individuals who did not modify their intake over a 15-year follow-up period, less severe disability outcomes were observed, particularly in females [70]. Moreover, the categorization of “heavy drinkers” based on Byrd’s criteria (more than one drink/day for females and more than two drinks/day for males) includes patients reporting an average of about 31 g of ethanol per day, which corresponds to two and a half servings per day. This level of consumption is slightly above recommendations, which cannot be considered indicative of ethanol abuse. Finally, considering the cross-sectional design of this study, it is not possible to certainly discern whether higher alcohol intake is causally related to disease severity or whether the results may depend on reverse causation [71,72]. Specifically, patients with less severe conditions may feel more comfortable with ethanol consumption. High physical activity was also associated with higher odds of mild MS. A previous study reported an inverse association between vigorous exercise and MS development, with a 27% greater odds of lower disability [73]. Moderate physical activity was also found to have a causal relationship with a reduced risk of MS in a Mendelian randomization study [74]. It is also possible that participants with lower disease severity could have been able to engage in high physical activity compared to those with moderate to high disease severity, due to reduced impairment.

The present study presents some limitations. The study design does not permit the retrieval of any causality between the observed associations. The COVID-19 pandemic required a self-assessment of the anthropometric data, possibly adding bias in the analysis. Due to law restrictions enforced during that particular period, this bias could not be avoided. To prevent the introduction of further biases, we performed a telephonic interview instead of using a questionnaire’s self-completion. Certainly, the reduced sample size and its geographical distribution limit the generalization of our findings. A greater number of participants from other regions would also have allowed us to extend the results to a wider population or investigate associations with other MS-related parameters (e.g., fatigue). Despite including a consistent number of confounding variables, we might have missed some (e.g., the absence of biochemical parameters). Moreover, the FFQ, DIS, and LIS used are not free from constraints. The addition of other instruments to evaluate dietary habits (e.g., food diary or recall) could have helped in better detailing nutritional intakes. Nevertheless, FFQ and DIS and LIS have some strengths. The FFQ used permitted us to investigate dietary habits in the medium term (6 months) instead of a limited time period (i.e., 3 or 7 days for the food diary and 1 day for the 24 h recall). Additionally, this specific FFQ was composed of 110 items, allowing us to assess a broader spectrum of items than those that could have been reported by other instruments. Moreover, being a semi-quantitative tool, the FFQ let us more precisely estimate the food quantities and, therefore, the nutritional composition of the participants’ habits. Further, this FFQ took into account foods’ seasonality. The DIS and LIS can be easily calculated from many FFQs and lifestyle questionnaires commonly used. Moreover, the use of both scores accounted for the contributions of diet and lifestyle to systemic inflammation. Mixed dishes or recipes were also included in the DIS analysis, making it more representative of the usual diets.

## 5. Conclusions

Although no differences were found in the total Dietary and Lifestyle Inflammatory Scores, some of their components might be connected with MS severity. Specifically, leafy green consumption, physical activity, and alcohol consumption may be related to MS severity. Further intervention studies are needed to prove our findings. Future studies should also examine the possible relationship between the pro- and anti-inflammatory capacities of dietary patterns and lifestyle factors on other MS-related parameters (e.g., fatigue).

## Figures and Tables

**Table 1:** Baseline sociodemographic and nutritional characteristics of sample (n = 107) according to DIS and LIS.

	DIS			LIS		
	Anti-Inflammatory (n = 53)	Pro-Inflammatory (n = 54)	*p*-Value	Anti-Inflammatory (n = 50)	Pro-Inflammatory (n = 57)	*p*-Value
Age, mean (SD)	51.6 (10.8)	49.0 (12.4)	0.249	50.0 (10.5)	50.5 (12.7)	0.823
BMI status, % (n)			0.913			0.000
Normal weight	67.9% (36)	64.8% (35)		88.0% (44)	47.4% (27)	
Overweight	26.4% (14)	27.8% (15)		12.0% (6)	40.4% (23)	
Obesity	5.7% (3)	7.4% (4)		0.0% (0)	12.3% (7)	
Sex, % (n)			0.784			0.023
Male	32.1% (17)	29.6% (16)		20.0% (10)	40.4% (23)	
Female	67.9% (36)	70.4% (38)		80.0% (40)	59.6% (34)	
Smoking habits, % (n)			0.638			0.063
Never	64.2% (34)	59.3% (32)		72.0% (36)	52.6% (30)	
Current smoker	15.1% (8)	22.2% (12)		10.0% (5)	26.3% (15)	
Former smoker	20.8% (11)	18.5% (10)		18.0% (9)	21.1% (12)	
Physical activity, % (n)			0.595			0.022
Low	41.5% (22)	38.9% (21)		28.0% (14)	50.9% (29)	
Moderate	28.3% (15)	37.0% (20)		34.0% (17)	31.6% (18)	
High	30.2% (16)	24.1% (13)		38.0% (19)	17.5% (10)	
Education, % (n)			0.552			0.331
Low	24.5% (13)	16.7% (9)		22.0% (11)	19.3% (11)	
Medium	45.3% (24)	53.7% (29)		42.0% (21)	56.1% (14)	
High	30.2% (16)	29.6% (16)		36.0% (18)	24.6% (14)	
MSSS, mean (SD)	3.3 (2.6)	2.9 (2.2)	0.372	3.2 (2.5)	3.1 (2.4)	0.764
Therapy, % (n)			0.633			0.736
No	35.8% (19)	31.5% (17)		32.0% (16)	35.1% (20)	
Yes	64.2% (34)	68.5% (37)		68.0% (34)	64.9% (37)	
Therapy type, % (n)			0.888			0.888
Moderate efficacy	73.5% (25)	75.0% (27)		73.5% (25)	75.0% (27)	
High efficacy	26.5% (9)	25.0% (9)		26.5% (9)	25.0% (9)	
MS phenotype, % (n)			0.628			0.449
Secondary progressive	28.3% (15)	20.4% (11)		26.0% (13)	22.8% (13)	
Relapsing–remitting	67.9% (36)	75.9% (41)		68.0% (34)	75.4% (43)	
Primary progressive	3.8% (2)	3.7% (2)		6.0% (3)	1.8% (1)	
Disease duration, mean (SD)	22.0 (12.3)	19.7 (11.3)	0.349	19.6 (11.4)	21.9 (12.3)	0.357

SD = standard deviation; BMI = body mass index; MSSS = Multiple Sclerosis Severity Score; MS = multiple sclerosis.

**Table 2:** Differences in energy and nutrient intakes based on DISs and LISs (n = 107).

Nutrient	DIS			LIS		
	Anti- Inflammatory (n = 53)	Pro- Inflammatory (n = 54)	*p*-Value	Anti- Inflammatory (n = 50)	Pro- Inflammatory (n = 57)	*p*-Value
Energy (kcal)	2005.0 (595.8)	1977.9 (572.2)	0.811	1998.9 (551.0)	1984.7 (611.7)	0.900
Energy (kJ)	8134.6 (2507.3)	8060.1 (2383.5)	0.875	8145.0 (2325.1)	8054.8 (2546.1)	0.849
Protein (g)	84.5 (28.1)	75.3 (22.7)	0.064	79.5 (21.5)	80.1 (29.3)	0.898
Lipids (g)	64.4 (28.2)	63.0 (19.4)	0.776	64.9 (23.9)	62.7 (24.3)	0.646
Cholesterol (mg)	164.8 (89.3)	160.6 (73.5)	0.791	152.9 (62.3)	171.4 (94.7)	0.243
SFA (%)	24.0 (13.6)	24.8 (8.5)	0.701	24.1 (9.6)	24.6 (12.6)	0.822
MUFA (%)	26.7 (10.9)	26.8 (7.6)	0.988	27.3 (9.7)	26.3 (9.1)	0.590
PUFA (%)	11.6 (5.9)	11.4 (4.6)	0.826	12.3 (6.0)	10.8 (4.5)	0.152
Carbohydrates (g)	284.7 (86.3)	285.4 (99.0)	0.967	288.6 (90.0)	282.0 (95.3)	0.716
Fiber (g)	35.1 (14.5)	26.3 (10.3)	<0.001	32.1 (10.7)	29.3 (15.2)	0.282
Vitamin A (µg)	1044.0 (472.2)	734.6 (271.8)	<0.001	889.9 (352.0)	886.1 (462.9)	0.962
Vitamin C (mg)	199.9 (93.7)	150.0 (86.5)	0.005	181.9 (77.1)	168.4 (105.5)	0.458
Vitamina E (mg)	10.1 (4.3)	8.1 (2.6)	0.004	9.6 (4.0)	8.6 (3.4)	0.196
Vitamin D (µg)	5.1 (3.9)	3.3 (2.0)	0.005	4.0 (2.7)	4.4 (3.5)	0.561
Vitamin B12 (µg)	5,6 (3.1)	4.7 (2.3)	0.088	4.8 (2.7)	5.5 (2.8)	0.253
Thiamin (mg)	1.9 (0.8)	1.6 (0.7)	0.051	1.8 (0.7)	1.7 (0.8)	0.727
Riboflavin (mg)	2.4 (1.0)	2.0 (0.7)	0.005	2.2 (0.8)	2.2 (1.0)	0.937
Niacin (mg)	23.1 (8.1)	20.0 (5.8)	0.028	21.6 (5.8)	21.5 (8.2)	0.982
Piridoxin (mg)	1380.4 (425.2)	1301.6 (401.4)	0.326	1335.4 (429.6)	1345.2 (402.3)	0.903
Folate (µg)	445.5 (180.8)	342.3 (112.3)	<0.001	339.4 (134.7)	371.1 (181.7)	0.369
Sodium (mg)	2086.0 (752.5)	2287.3 (725.3)	0.162	2125.0 (618.9)	2242.6 (837.3)	0.416
Potassium (mg)	4081.4 (1311.5)	3199.8 (1099.0)	<0.001	3721.6 (1118.4)	3561.8 (1415.9)	0.523
Iron (mg)	16.2 (6.1)	13.1 (4.5)	0.003	15.1 (4.8)	14.3 (6.2)	0.454
Calcium (mg)	911.2 (449.6)	759.0 (290.2)	0.046	831.7 (336.0)	836.8 (443.6)	0.947
Phosphorus (mg)	1472.3 (523.7)	1253.7 (385.8)	0.015	1379.5 (415.3)	1346.6 (516.6)	0.720
Magnesium (mg)	450.6 (159.8)	371.3 (121.1)	0.005	423.0 (127.3)	399.7 (161.7)	0.415
Zinc (mg)	12.2 (4.4)	10.3 (3.3)	0.016	11.2 (3.4)	11.3 (4.4)	0.933
Copper (mg)	2.3 (0.9)	1.8 (0.7)	0.005	2.1 (0.7)	2.0 (0.9)	0.371
Selenium (µg)	98.6 (41.2)	99.3 (39.2)	0.931	96.3 (34.5)	101.2 (44.5)	0.535

DIS = dietary inflammatory score; LIS = lifestyle inflammatory score; SFA = saturated fatty acids; MUFA = monounsaturated fatty acids; PUFA = polyunsaturated fatty acids.

**Table 3:** Association between major DIS components and mild Multiple Sclerosis Severity Scores compared to moderate-to-high scores (n = 92).

Variable	Multiple Sclerosis Severity, OR (95% CI)	
	Moderate to High	Mild
Leafy greens and cruciferous vegetables	1	1.67 (1.50, 18.74)
Tomatoes	1	0.08 (0.33, 3.56)
Apples and berries	1	0.44 (0.52, 4.55)
Deep yellow or orange vegetables and fruits	1	0.61 (0.56, 5.99)
Other fruits and real fruit juices	1	0.67 (0.16, 1.66)
Other vegetables	1	0.81 (0.13, 1.54)
Legumes	1	0.28 (0.26, 2.24)
Fish	1	0.12 (0.37, 3.43)
Poultry	1	0.53 (0.17, 1.97)
Red and organ meats	1	0.60 (0.19, 1.55)
Processed meats	1	0.06 (0.37, 3.06)
Added sugars	1	0.53 (0.61, 4.76)
High-fat dairy	1	0.14 (0.31, 2.41)
Low-fat dairy	1	0.31 (0.44, 4.24)
Coffee and tea	1	0.24 (0.27, 2.26)
Nuts	1	0.97 (0.12, 1.16)
Other fats	1	0.61 (0.61, 5.50)
Refined grains and starchy vegetables	1	0.62 (0.48, 3.98)

**Table 4:** Association between major LIS components and mild Multiple Sclerosis Severity Score compared to moderate-to-high scores (n = 92).

Variable	Multiple Sclerosis Severity, OR (95% CI)	
	Moderate to High	Mild
Heavy drinker	1	5.61 (1.19, 26.47)
Moderate drinker	1	1.22 (0.44, 3.33)
Moderate physical activity	1	2.07 (0.67, 6.42)
High physical activity	1	5.51 (1.66, 18.30)
Current smoker	1	1.21 (0.32, 4.51)
Overweight	1	1.45 (0.51, 4.11)
Obesity	1	6.38 (0.94, 43.48)

## Data Availability

The data presented in this study are available on request from the corresponding author due to privacy reasons.
